# Community Cardiac Rehabilitation Program: Lessons Learned for Long‐Term Outcomes

**DOI:** 10.1111/wvn.70089

**Published:** 2025-12-04

**Authors:** Cheryl Monturo, Carol Smith, William M. Bannon, Cindy Brockway

**Affiliations:** ^1^ Chester County Hospital Penn Medicine West Chester Pennsylvania USA; ^2^ William Bannon Associates, Inc New York New York USA

**Keywords:** behavioral, cardiac event, cardiac rehabilitation, diet, exercise, long‐term outcomes

## Abstract

**Background:**

Although some research supports the maintenance of positive outcomes from cardiac rehabilitation Phase II (CR II) up to 12 months, the barriers to maintaining physical activity and risk factor management during CR maintenance (CR III) are well known.

**Aim:**

To investigate participants' ability to sustain clinical, quality‐of‐life (QOL), and behavioral outcomes and share their experiences 6 months after CR II completion.

**Methods:**

Longitudinal explanatory sequential pre‐CR/post‐CR study in a community hospital within a large health system. A convenience sample of 155 participants completed a reassessment of health outcomes. Participants also completed an online survey about barriers and facilitators during and after CR II. Analysis methods included MANOVA and summative content analysis.

**Results:**

The sample was mostly male, white, non‐Hispanic, and college educated, with a mean age of 67.9 years. CR II participants sustained most behavioral outcomes, but not all clinical outcomes. Outcomes that improved/maintained were physical activity, tobacco status, diet, and QOL. Outcomes that worsened/returned to pre‐CR II were weight, blood pressure, and depression. Participants described their motivation for staying healthy, top concerns, goals, barriers, and strengths/resources. Common responses included exercise, weight, diet, quality of life, family, and friends.

**Linking Evidence to Action:**

Our findings suggest the need for the implementation of innovative strategies during CR II that may extend past discharge into CR III. These include the introduction of digital technology and eHealth to provide value‐added service to patients and a solid foundation for future maintenance and a structured, behavioral weight loss intervention. Establishing these tools, in addition to developing a support system will help patients to initiate maintenance care before program completion.

## 1 Background and Significance

Cardiac rehabilitation phase II (CR II) is a secondary prevention program designed to improve health after a cardiac event and mitigate the risk of a second event. In a recent Cochrane Review, Dibben et al. (2023) confirmed the results of an earlier review (Andersen et al. 2016) reporting continued positive outcomes of cardiac rehabilitation (CR) for up to 12 months. Outcomes included reduced risk of myocardial infarction (MI), a small reduction in all‐cause mortality, a large decrease in all‐cause hospitalization and healthcare costs, and improved healthcare‐related quality of life (Dibben et al. 2023). Additionally, others reported improved heart‐healthy diet intake (Laursen et al. 2021), and a likely reduction in heart failure‐specific hospital admissions in the short term (up to 12 months) (Long et al. 2019).

With continuous observation from the clinical team and 80% of the costs covered by Medicare, participants are motivated and accountable to maintain goals set in CR II. Conversely, phase III maintenance (CR III) is characterized by self‐regulation, often a lack of insurance coverage, and a lack of support to maintain their goals. Some CR programs offer extended outpatient self‐pay services including supervised exercise, but not a comprehensive physiological and psychological reassessment that occurs after CR II program discharge (Cardiovascular Mayo Clinic 2024).

To address these barriers, researchers have used innovative methods for follow‐up including a smartphone app that improved quality‐of‐life outcomes, maximal oxygen consumption, exercise performance, exercise habits, and self‐perceived goal achievement at 1 year (Lunde et al. 2020). Others reported the effectiveness of electronic communication and health information technology (eHealth) on promotion of physical activity and exercise capacity at 6 months (Heimer et al. 2023). In contrast, participants in a smartphone and social media‐based CR program reported no difference in quality‐of‐life outcomes at 6 months post CR discharge as compared to those not in a CR program (Dorje et al. 2019). This variability in findings may be due to the potential risk of bias, heterogeneity, and small sample size in phase III studies negatively impacting the quality of evidence (Heimer et al. 2023; Long et al. 2019). Although some innovative models effectively replaced center‐based CR during the pandemic (Yang et al. 2023) and were supported through temporary funding (CMS 2023), these are no longer supported.

The purpose of this study was to investigate patients' ability to sustain clinical, behavioral, and quality of life outcomes 6 months after cardiac rehabilitation program discharge. Primary aims were (1) to investigate whether patients can sustain changes in weight, BMI, waist circumference, resting blood pressure, functional capacity, exercise program, and tobacco/smoking status through self‐regulation, (2) to investigate whether patients can sustain changes in quality of life, depression, nutrition habits, and medication compliance through self‐regulation, and (3) to examine patient experiences after CR program completion which served as barriers or facilitators to program adherence during and after the program.

## 2 Methods

### 2.1 Design and Setting

This longitudinal explanatory sequential pre‐CR/post‐CR study evaluated the effect of CR at three distinct time points (pre‐CR, post‐CR, follow‐up) during the COVID‐19 pandemic (2020–2022) and examined the barriers and facilitators to CR program adherence. After institutional review board approval, the study was conducted at an outpatient multidisciplinary medically supervised cardiopulmonary program affiliated with a suburban community hospital, which is part of a large regional health system. Up to 36 visits over several months are usually covered by insurance and most participants attend the majority once they begin the program. Upon discharge, there is no formal long‐term evaluation through the program; instead they are referred to a cardiologist for follow‐up.

### 2.2 Sample

G*power 3.1.9.4 statistical analysis was used to determine sample size. The software indicated that a within‐factors repeated measures general linear model with three timepoints, with alpha set at 0.05, power at 0.8, a correlation among repeated measures of 0.1, and nonsphericity correlation of 1, would detect a small effect size (*f* = 0.14) with a sample size of 149 study participants. Subsequently, the CR nurse recruited a convenience sample of 155 patients who completed the program approximately 6 months prior. Patients were referred through external resources as well as the affiliated hospital, attended a minimum of 18 sessions, and successfully graduated from the program.

### 2.3 Measures

Researchers used the same assessment tools from the patient's admission and discharge from CR. Measurements included height, weight, BMI, waist circumference, resting blood pressure, functional capacity (6‐min walk test [6MWT]), a prescribed exercise program (minutes/week [METs]) and tobacco/smoking status determination. Surveys included Dartmouth Quality of Life, PHQ9 Depression Scale, RYP‐Rate your Plate nutritional habits, and medication compliance. Reassessment was at no cost to the patient. Based on these quantitative tools and the responses of participants, the team developed a brief qualitative survey reflecting broader heart health goals, some of which identified key factors that support adherence to treatment plans (Rashidi et al. 2020). The survey included five open‐ended questions:

1. What is your motivation for staying healthy?
2. What goals have you made (i.e., physical activity, diet, etc.) following discharge from cardiac rehabilitation?
3. Since your discharge from cardiac rehabilitation, what has been your top concern?
4. What are personal, social (i.e., family roles), or environmental barriers to staying healthy?
5. What are personal strengths, social or community resources that you use to stay healthy? The survey was distributed via email after completion of the in‐person reassessment.

### 2.4 Data Management and Analysis

Statistical analyses for quantitative data were performed using SPSS version 29 (IBM SPSS Statistics for Windows, IBM Corp., Armonk, NY, USA). A multivariate model, MANOVA, was used to model changes over time. Each model was assessed in terms of overall statistical significance and partial eta squared (PES) effect size values. All test assumptions were observed, including checks of sphericity. Due to the brevity of survey responses, researchers used summative content analysis through SPSS version 29. This analysis included the identification and quantification of the most commonly occurring words in addition to discovering the underlying meaning of the words, or latent content analysis (Hsieh and Shannon 2005).

## 3 Results

The convenience sample of 155 subjects was mostly male (*n* = 114, 73.5%), white (*n* = 144, 92.9%), non‐Hispanic (*n* = 149, 96.1%) and reported an educational level of ≥ 4 years college (*n* = 87, 56.1%). The average age for the sample was 67.90 years (SD = 9.97, Min/Max = 29–90). One hundred and sixty‐four additional patients were invited to participate, but refused, did not show up for the appointment, or did not respond to voicemail messages. Almost half (*n* = 71, 45.8%) of the subjects, initiated rehab because of a percutaneous coronary intervention (PCI) (Table 1). Demographics (gender, ethnicity, race, or age) had no impact on clinical, behavioral, or quality of life outcomes.

**TABLE 1:** Descriptive analysis of the demographic study participant characteristics (*n* = 155).

Variable	*N*	%
Diagnosis (all that apply)		
Valve related	48	31.0
Coronary artery bypass graft	25	16.1
Drug‐eluting stent (DES)/percutaneous	71	45.8
Coronary intervention (PCI)		
Heart failure	5	3.2
Heart transplant	4	2.6
Non‐ST elevation MI	19	12.3
ST elevation MI	5	3.2
Stable angina	4	2.6
Multiple diagnoses	26	16.8
Gender		
Female	41	26.5
Male	114	73.5
Race		
White	144	92.9
African–American/Black	9	5.8
Asian	2	1.3
Ethnicity		
Hispanic	6	3.9
Non‐Hispanic	149	96.1
Education		
≥ 8th Grade	3	1.9
High School Degree	33	21.3
Some college/technical school	32	20.6
≥ 4 years college	87	56.1
Education (collapsed for inferential analysis)		
Less than 4‐year college degree	68	43.9
≥ 4 years college	87	56.1
Age	*M* = 67.90, SD = 9.97, Min/Max = 29–90	

### 3.1 Quality of Life Variables

Subjects maintained improvement in all quality‐of‐life variables at follow‐up, except in pain (*M* = 2.26, SD = 1.15), *p* = 0.10 and change in health (*M* = 2.26, SD = 0.91), *p* < 0.01 (Table 2 and Table S1). Subjects reported a positive change in health post‐CR (*M* = 1.52, SD = 0.75), *p* < 0.05, but follow‐up scores showed a significant reversal in that trend with a more negative change to their health (*M* = 2.26, SD = 0.91), *p* < 0.001. Reports on social support revealed no statistically significant changes in any direction across the three time points (Table 2 and Table S1).

**TABLE 2:** Repeated measures MANOVA examining quality of life outcome variables (*n* = 155).

Variable	*n*	*M* (SD)	*F* (df)	*p*	PES
Physical fitness			88.32 (1.90, 229.34)	0.001	0.36^1^
Pretest	155	3.60 (1.09)			
Posttest	155	2.50 (1.09)			
Follow‐up	155	2.45 (1.17)			
Feelings			15.30 (1.81, 279.19)	0.001	0.09^2^
Pretest	155	2.01 (1.05)			
Posttest	155	1.61 (0.87)			
Follow‐up	155	1.74 (0.91)			
Daily activities			60.57 (1.69, 260.80)	0.001	0.28^3^
Pretest	155	2.41 (1.11)			
Posttest	155	1.62 (0.86)			
Follow‐up	155	1.61 (0.84)			
Social activities			37.64 (1.64, 252.34)	0.001	0.20^4^
Pretest	155	2.05 (1.17)			
Posttest	155	1.41 (0.84)			
Follow‐up	155	1.38 (0.76)			
Pain			3.61 (1.89, 291.42)	0.03	0.02^5^
Pretest	155	2.32 (1.08)			
Posttest	155	2.08 (0.96)			
Follow‐up	155	2.26 (1.15)			
Change in health			33.55 (1.90, 292.30)	0.001	0.18^6^
Pretest	155	1.77 (0.97)			
Posttest	155	1.52 (0.75)			
Follow‐up	155	2.26 (0.91)			
Overall health			27.15 (1.87, 287.30)	0.001	0.15^7^
Pretest	155	2.78 (0.86)			
Posttest	155	2.32 (0.81)			
Follow‐up	155	2.33 (0.93)			
Social support			0.67 (1.98, 304.95)	0.51	0.004^8^
Pretest	155	1.46 (0.80)			
Posttest	155	1.43 (0.83)			
Follow‐up	155	1.52 (0.96)			
Quality of life			25.67 (1.93, 296.59)	0.001	0.14^9^
Pretest	155	2.10 (0.65)			
Posttest	155	1.73 (0.64)			
Follow‐up	155	1.79 (0.73)			

*Note:* The significance for superscripts is related to supplemental files.

### 3.2 Clinical Variables

Subjects demonstrated a statistically significant increase in the number of feet covered in a 6MWT at follow‐up as compared to their post‐CR scores (*M* = 1, 801.60, SD = 467.58), *p* < 0.001, and maintained a decrease in waist circumference (*M* = 39.82, SD = 5.41), *p* = 1.00 (Table 3 and Table S2). However, systolic BP (SBP) (*M* = 121.01, SD = 13.84), *p* < 0.001, diastolic BP (DBP) (*M* = 68.03, SD = 9.75), *p* < 0.001, the presence of depression (*M* = 2.66, SD = 3.74), *p* < 0.05, BMI (*M* = 28.72, SD = 4.73), *p* < 0.05, and weight in pounds (*M* = 189.29, SD = 36.92), *p* < 0.05 increased at statistically significant levels at follow‐up. There were no statistically significant differences in patients receiving statin therapy across all time points (*F* [1.72, 265.40] =.87, *p* = 0.41) (Table 3 and Table S2).

**TABLE 3:** Repeated measures MANOVA examining clinical outcome variables (*n* = 155).

Variable	*n*	*M* (SD)	*F* (df)	*p*	PES
Feet covered in a 6‐min walk			320.73 (1.45, 218.14)	0.001	0.68^1^
Pretest	152	1,308.84 (388.65)			
Posttest	152	1,746.88 (451.36)			
Follow‐up	152	1,801.60 (467.58)			
Percentage of patients on statins now			0.87 (1.72, 265.40)	0.41	0.006^2^
Pretest	155	0.72 (0.45)			
Posttest	155	0.74 (0.44)			
Follow‐up	155	0.75 (0.44)			
Weight in pounds			4.00 (1.54, 235.518)	0.03	0.03^3^
Pretest	154	187.90 (36.62)			
Posttest	154	187.63 (36.03)			
Follow‐up	154	189.29 (36.92)			
Body mass index			3.64 (1.50, 229.85)	0.03	0.02^4^
Pretest	154	28.53 (4.89)			
Posttest	154	28.47 (4.70)			
Follow‐up	154	28.72 (4.73)			
Waist circumference (in inches)			11.45 (1.88, 257.824)	0.001	0.08^5^
Pretest	138	40.39 (5.28)			
Posttest	138	39.72 (5.01)			
Follow‐up	138	39.82 (5.41)			
Depression screening			19.90 (1.64, 251.87)	0.001	0.11^6^
Pretest	155	3.91 (4.06)			
Posttest	155	2.08 (2.91)			
Follow‐up	155	2.66 (3.74)			
Systolic BP			16.57 (1.94, 298.00)	0.001	0.10^7^
Pretest	155	120.09 (14.33)			
Posttest	155	114.80 (10.93)			
Follow‐up	155	121.01 (13.84)			
Diastolic BP			24.18 (1.92, 294.94)	0.001	0.14^8^
Pretest	155	69.38 (8.24)			
Posttest	155	64.36 (6.95)			
Follow‐up	155	68.03 (9.75)			

*Note:* The significance for superscripts is related to supplemental files.

### 3.3 Behavioral Variables

Subjects maintained positive behavioral outcomes in making healthier dietary choices (*M* = 59.33, SD = 6.53), *p* = 0.17; smoking less tobacco (*M* = 0.03, SD = 0.18), *p* = 0.96; and needing tobacco smoking interventions (*M* = 0.03, SD = 0.18), *p* = 1.0 at follow‐up (Table 4 and Table S3). Subjects' physical activity in METs (*M* = 689.74, SD = 584.08), *p* < 0.01, and medication habits (*M* = 6.91, SD = 0.31), *p* < 0.001 decreased significantly at follow‐up from post‐CR (Table 4 and Table S3).

**TABLE 4:** Repeated measures MANOVA examining behavioral outcome variables (*n* = 155).

Variable	*n*	*M* (SD)	*F* (df)	*p*	PES
Rate my plate (dietary habits)			39.27 (1.85, 284.70)	0.001	0.20^1^
Pretest	155	56.44 (6.93)			
Posttest	155	60.02 (6.62)			
Follow‐up	155	59.33 (6.53)			
Physical activity habits in MET minutes per week			121.47 (1.87, 285.328)	0.001	0.44^2^
Pretest	154	250.99 (359.85)			
Posttest	154	807.31 (470.74)			
Follow‐up	154	689.74 (584.08)			
Medication habits (prescription adherence: # days of 7)			6.31 (1.73, 267.01)	0.003	0.04^3^
Pretest	155	6.92 (0.32)			
Posttest	155	7.00 (0.00)			
Follow‐up	155	6.91 (0.31)			
Tobacco smoking intervention (% yes)			11.85 (1.28, 196.842)	0.001	0.07^4^
Pretest	155	0.11 (0.31)			
Posttest	155	0.03 (0.18)			
Follow‐up	155	0.03 (0.18)			
Tobacco/smoking habits (% using tobacco/smoking)			7.42 (1.21, 184.56)	0.005	0.05^5^
Pretest	154	0.08 (0.27)			
Posttest	154	0.03 (0.16)			
Follow‐up	154	0.03 (0.18)			

*Note:* The significance for superscripts is related to supplemental files.

### 3.4 Qualitative Open‐Ended Questions

Out of the original 155 subjects, 89 study participants provided qualitative data. However, 4 are not represented in the table (Table 5) due to missing data. Of the 85 study participants that provided qualitative data and were matched with their demographic information, the subjects mirrored the full sample of 155 subjects in gender, race, ethnicity, education, and age (Table 5), and these had no impact on clinical, behavioral, or QOL outcomes. Summative content analysis of responses was initially organized by question and then categorized into two broad themes: facilitators and barriers. Facilitators included the categories of participant motivation, goals, and resources. Barriers included the category of top concerns. The top three frequency counts for each category are recorded in Table 6.

**TABLE 5:** Descriptive analysis of the demographic study participant characteristics (*n* = 85).^a^

Variable	*N*	%
Diagnosis (all that apply)		
Valve related	28	32.9
Coronary artery bypass graft	15	17.6
Drug‐eluting stent (DES)/percutaneous	37	43.5
Coronary intervention (PCI)		
Heart failure	3	3.5
Heart transplant	1	1.2
Non‐ST elevation MI	9	10.6
ST elevation MI	4	4.7
Stable angina	4	2.7
Multiple diagnoses	16	18.8
Gender		
Female	28	32.9
Male	57	67.1
Race		
White	80	94.1
African–American/Black	5	5.9
Ethnicity		
Hispanic	0	0.0
Non‐Hispanic	85	100.0
Education		
≥ 8th Grade	0	0.0
High School Degree	18	21.2
Some college/technical school	18	21.2
≥ 4 years college	48	56.5
Education (collapsed for inferential analysis)		
Less than 4 year college degree	37	43.5
≥ 4 years college	48	56.5
Age	*M* = 67.76, SD = 8.74, Min/Max = 47–88	

^a^ Of the study participants that provided qualitative data (*n* = 89), 4 are not represented in this table.

**TABLE 6:** Most common qualitative themes and categories.^a^

Facilitators						Barriers			
Motivation		Goals		Resources		Top concerns		Barriers	
Variable	*N*/%	Variable	*N*/%	Variable	*N*/%	Variable	*N*/%	Variable	*N*/%
Enhanced quality of life	49/55	Exercise related	72/81	Family	24/27	General health, staying active	25/28	None	26/29
Staying alive, longevity	27/30	Diet related	45/51	Self‐determination, motivation	23/26	Maintaining exercise^b^	12/14	Self‐motivation, not being lazy	15/17
Family and friends related	26/29	Body weight related^b^	17/19	Exercise, available exercise areas	23/26	Avoid recurrence, new problems^b^	12/14	Issues that challenge diet	14/16
		Maintain general health, well‐being, and/or to manage stress^b^	17/19			A specific condition, symptom	11/12		

^a^ Percentages rounded to nearest whole number for display.

^b^ Tied.

#### 3.4.1 Facilitators

Participants were asked to identify the motivation for staying healthy, their goals, and the available resources to reach these goals. Participants described their motivation to enhance quality of life (55.1%) as “To be able to do the things I want to do,” “To enjoy life,” or “It's not fun to be unhealthy.” The possibility of a longer life motivated many participants (30.3%) to stay healthy, hoping to live for “90 years” or “to live past 100.” Participants combined this motivation with family and friend‐related goals (29.2%) in “living a long life to spend with my family,” or “living a long life for my husband and children.”

Participant goals focused on exercise (80.9%), diet (50.6%), body weight (19.1%), and maintenance of general health/well‐being, and/or to manage stress (19.1%). Exercise‐related responses included general plans to “keep exercising on schedule…” or “more exercise.” Diet‐related responses were less granular but broadly identified the dos and don'ts such as yes to veggies, fish, chicken, and fruit and no to alcohol, red meat, and fatty foods. Some participants specified a maximum goal of “200 lbs.” or weight loss accomplishments, “already lost 40 pounds” or plans “I need to lose another 10….” The last major goal, to manage stress, seemed to combine much of the first three: exercise, diet, and weight management in a more generic approach to health, stress reduction, maintenance of blood pressure, and rest.

Participants described the way in which they used personal strengths and social or community resources to stay healthy. These included family (27%), self‐determination/motivation (25.8%), and exercise/available exercise areas (25.8%). Family included “wives, pets, and children.” Self‐determination/motivation often overlapped with exercise identifying programs such as “Silver Sneakers” and the “YMCA,” in addition to diet‐related quotes such as “I refrain from bad foods…”

#### 3.4.2 Barriers

A considerable number of respondents reported that they had no personal, social, or environmental barriers to staying healthy (29.2%) saying “Nothing will stop me,” “Retired – no barriers,” and simply “None.” Participants also reported that self‐motivation or “not being lazy” (16.9%) and issues that challenged their diet (15.7%) were also barriers. One participant connected a diet and work‐related barrier: “My job in running a business has little down time…so lots of stress, hard to control whether I can eat lunch or not…” Finally, one participant identified themselves as a potential barrier, simply responding “me.”

Barriers also included a long and detailed list of top concerns since discharge from CR with general health/staying active as the most commonly occurring response (28.1%). Participants identified other most commonly occurring concerns such as the need to maintain exercise routines (13.5%) despite “COVID gym lockdowns,” the possibility of exacerbation of their cardiac condition or the appearance of “new problems” (13.5%), and the occurrence of a specific condition or symptom (12.4%) such as “leg weakness” and “more fatigue than I'd like.”

## 4 Discussion

The barriers to maintaining physical activity and risk factor management are well documented (Graham et al. 2020; Yates et al. 2017). In this study, participants discharged from CR II were referred to different cardiologists with varying timelines for follow‐up, and not to a formal CR III maintenance program. Notwithstanding the lack of standardization, participants maintained or improved in 2/3 of their quality‐of‐life outcomes at follow‐up consistent with previous reports of up to 12 months (Dibben et al. 2023). They often attributed their success to resources such as personal strength and the impact of friends/family, consistent with previous research (Keenan 2017; Costa et al. 2015). Despite this attribution participants reported no significant changes in social support across all 3 time points in the Dartmouth Quality‐of‐Life Scale. Perhaps they highlighted the importance of family and friends as their ideal but failed to report that this remained an unmet need. Of note, participants commented to the CR nurse during the follow‐up reevaluation that they were suffering from loneliness; however, they did not report this in the qualitative survey.

Participants in this study maintained healthier dietary choices at follow‐up, yet previous research reported variable findings (Breitinger et al. 2021; Ghisi et al. 2021; Laursen et al. 2021). Although participants maintained a healthy diet, they also reported experiencing challenges. It is likely that these challenges were a factor in their inability to maintain weight loss, instead showing a statistically significant weight increase at follow‐up. This finding is consistent with literature highlighting the difficulty in making lifestyle changes (Rashidi et al. 2020) including weight loss. More recent literature suggests the addition of a structured, behavioral weight loss intervention to an exercise‐based CR rehabilitation program (Brinkley et al. 2023).

Although participants did not report complete cessation of smoking, they did smoke tobacco less at follow‐up. This improvement is consistent with observational research stating that smokers participating in CR (outpatient) were more likely to quit smoking than those who did not attend (Katz et al. 2019).

Despite evidence of positive trends in both behavioral and quality‐of‐life outcomes, participants in this study only sustained improvement in two out of eight clinical outcomes: the 6‐min walk (6MWT) and the waist circumference measurement. Participants walked further (*p* < 0.001) during the 6MWT at post‐CR and again at follow‐up. This test for assessing functional capacity is simple to use, inexpensive, and widely used to assess the effectiveness of a CR program (Hamilton and Haennel 2000). Decreases in waist circumference (inches) measurements from pre‐CR to post‐CR were also maintained at follow‐up, consistent with current research (Janssen et al. 2014).

Work has been cited as an inhibitory factor to maintaining exercise regimens (Rashidi et al. 2020) and balancing lifestyle changes particularly when returning to work after CR (Stendardo et al. 2018). Consistent with these findings, participants in this study identified balancing health and work as barriers or top concerns.

Progress made during CR was not maintained at follow‐up in medication adherence, SBP and DBP. Adherence to new medication regimens can be challenging, particularly for those taking five or more long‐term medications. Consistent with current literature, participants in this study demonstrated a statistically significant improvement in medication adherence at post‐CR (Gebremichael et al. 2024); however, they were unable to sustain that change at follow‐up, instead returning to pre‐CR status. Statistically significant decreases in SBP and DBP from pre‐CR to post‐CR were erased at follow‐up, with resultant increases in both (*p* < 0.001). In comparison, Janssen et al. (2014) demonstrated a statistically significant decrease in SBP from pre‐CR to follow‐up at 6 months.

Physical activity habits measured in METs were also not maintained at follow‐up; however, outcomes were still higher than pre‐CR findings (250–689 METs/week) and within the recommended range of 500–1000 MET min/week of moderate to vigorous physical activity (Eckel et al. 2014).

### 4.1 Strengths and Limitations

The qualitative survey response in this study was impressive, with almost 58% of the total sample participating. Demographically, this group did not differ from the total sample. Overall findings in this study may be limited by potential self‐selection bias, lack of randomization, and a long recruitment period to obtain the target sample size as a result of the pandemic. However, if self‐selection caused those who were doing well to participate, questions arise about the remainder who did not participate.

Further, due to institutional restrictions on research‐related in‐person time during the pandemic, initial plans for a focus group discussion over a hearty‐healthy meal were forfeited in lieu of an email survey using a semi‐structured guideline, limiting the depth of qualitative research findings. Future longitudinal studies including focus group discussions at several time points may encourage in‐depth exploration of the barriers to sustaining progress made during the CR II phase.

## 5 Linking Evidence to Action

Prior to completion of the CR II program:

- Guide participants in the creation of long‐term strategies, a support system, and early initiation of maintenance care.
- Advise participants of the potential rebound in clinical, behavioral, and quality of life outcomes after phase CR II completion.
- Introduce digital technology to provide a value‐added service and solidify patients' familiarity with eHealth and strategies to maintain positive outcomes and motivation during CR III.
- Emphasize the use of home monitoring with wearable technology, scales, and BP monitors during CR II and through CR III.
- Add a structured, behavioral weight loss intervention to the CR II program for a period of six months.
- Explore the potential for a patient run support group to address the gap in social support during CR III.

## 6 Conclusions

The barriers to maintaining physical activity and risk factor management after cardiac rehabilitation are well documented (Graham et al. 2020; Yates et al. 2017). Results of this study reinforce previous findings and recognize the likelihood that formal CR III maintenance programs are not the answer given the lack of funding and infrastructure. Despite the challenges experienced because of the pandemic, perhaps innovative programs using digital technology can enhance the link to CR II programs and partner with individual cardiology practices to improve patients' overall health.

## Conflicts of Interest

The authors declare no conflicts of interest.

## Supporting information


**Table S1:** wvn70089‐sup‐0001‐TablesS1‐S3.docx.

## Data Availability

The data that support the findings of this study are available on request from the corresponding author. The data are not publicly available due to privacy or ethical restrictions.
