# Supplementary material for: Community Cardiac Rehabilitation Program: Lessons Learned for Long‐Term Outcomes
Source: Worldviews Evid Based Nurs. 2025 Dec 4;22(6):e70089. doi: 10.1111/wvn.70089 (PMC12676506; doi:10.1111/wvn.70089)
Supplement: Supplementary file 1 — Table S1: wvn70089‐sup‐0001‐TablesS1‐S3.docx. [file WVN-22-0-s001.docx]

**Supplementary Table 1**

**Repeated Measures MANOVA Examining Quality of Life Outcome Variables (*n*=155)**

¹Bonferroni Pairwise Comparisons indicated that: 1) the pretest score was significantly higher than the posttest score (*p*<.001) and follow-up score (*p*<.001); 2) the posttest score was significantly lower than the pretest score (*p*<.001), but did not differ significantly from the follow-up score (*p*=1.00); and 3) the follow-up score was significantly lower than the pretest score (*p*<.001), but did not differ significantly from the posttest score (*p*=1.00).

²Bonferroni Pairwise Comparisons indicated that: 1) the pretest score was significantly higher than the posttest score (*p*<.001) and follow-up score (*p*<.01); 2) the posttest score was significantly lower than the pretest score (*p*<.001), but did not differ significantly from the follow-up score (*p*=.08); and 3) the follow-up score was significantly lower than the pretest score (*p*<.01), but did not differ significantly from the posttest score (*p*=.08).

^3^Bonferroni Pairwise Comparisons indicated that: 1) the pretest score was significantly higher than the posttest score (*p*<.001) and follow-up score (*p*<.001); 2) the posttest score was significantly lower than the pretest score (*p*<.001), but did not differ significantly from the follow-up score (*p*=1.00); and 3) the follow-up score was significantly lower than the pretest score (*p*<.001), but did not differ significantly from the posttest score (*p*=1.00).

^4^Bonferroni Pairwise Comparisons indicated that: 1) the Pretest score was significantly higher than the posttest score (*p*<.001) and follow-up score (*p*<.001); 2) the posttest score was significantly lower than the pretest score (*p*<.001), but did not differ significantly from the follow-up score (*p*=1.00); and 3) the follow-up score was significantly lower than the pretest score (*p*<.001), but did not differ significantly from the posttest score (*p*=1.00).

^5^Bonferroni Pairwise Comparisons indicated that: 1) the pretest score was significantly higher than the posttest score (*p*<.05), but did not differ significantly from the follow-up score (*p*=1.00); 2) the posttest score was significantly lower than the pretest score (*p*<.05), but did not differ significantly from the follow-up score (*p*=.10); and 3) the follow-up score did not differ significantly from the pretest score (*p*=1.00), as well as the posttest score (*p*=.10).

^6^Bonferroni Pairwise Comparisons indicated that: 1) the pretest score was significantly higher than the posttest score (*p*<.05), but was significantly lower than the follow-up score (*p*<.001); 2) the posttest score was significantly lower than the pretest score (*p*<.05), as well as the follow-up score (*p*<.001); and 3) the follow-up score was significantly higher than the pretest score (*p*<1.00), as well as the posttest score (*p*<.001).

^7^Bonferroni Pairwise Comparisons indicated that: 1) the pretest score was significantly higher than the posttest score (*p*<.001), as well as the follow-up score (*p*<.001); 2) the posttest score was significantly lower than the pretest score (*p*<.001), but did not differ significantly from the follow-up score (*p*=1.00); and 3) the follow-up score was significantly lower than the pretest score (*p*<.001), but did not differ significantly from the posttest score (*p*=1.00).

^8^Bonferroni Pairwise Comparisons: NA.

^9^Bonferroni Pairwise Comparisons indicated that: 1) the pretest score was significantly higher than the posttest score (*p*<.001), as well as the follow-up score (*p*<.001); 2) the posttest score was significantly lower than the pretest score (*p*<.001), but did not differ significantly from the follow-up score (*p*=.77); and 3) the follow-up score was significantly lower than the pretest score (*p*<.001), but did not differ significantly from the posttest score (*p*=77).

**Supplementary Table 2**

**Repeated Measures MANOVA Examining Clinical Outcome Variables (*n*=155)**

¹Bonferroni Pairwise Comparisons indicated that: 1) the pretest score was significantly lower than the posttest score (*p*<.001) and follow-up score (*p*<.001); 2) the posttest score was significantly higher than the pretest score (*p*<.001) and lower than the follow-up score (*p*<.001); and 3) the follow-up score was significantly higher than the pretest score (*p*<.001) and posttest score (*p*<.001).

²Bonferroni Pairwise Comparisons: NA

^3^Bonferroni Pairwise Comparisons indicated that: 1) the pretest score was not significantly different from the posttest score (*p*=1.00) and follow-up score (*p*=.23); 2) the posttest score was not significantly different than the pretest score (*p*=1.00), but was significantly lower than the follow-up score (*p*<.05); and 3) the follow-up score was not significantly different than the pretest score (*p*=.23), but was significantly higher than the posttest score (*p*<.05).

^4^Bonferroni Pairwise Comparisons indicated that: 1) the pretest score was not significantly different from the posttest score (*p*=1.00) and follow-up score (*p*=.35); 2) the posttest score was not significantly different than the pretest score (*p*=1.00), but was significantly lower than the follow-up score (*p*<.05); and 3) the follow-up score was not significantly different than the pretest score (*p*=.35), but was significantly higher than the posttest score (*p*<.05).

^5^Bonferroni Pairwise Comparisons indicated that: 1) the pretest score was significantly higher than the posttest score (*p*<.001) and follow-up score (*p*<.01); 2) the posttest score was significantly lower than the pretest score (*p*<.001), but did not differ significantly from the follow-up score (*p*=1.00); and 3) the follow-up score was significantly lower than the pretest score (*p*<.01), but did not differ significantly from the posttest score (*p*=1.00).

^6^Bonferroni Pairwise Comparisons indicated that: 1) the pretest score was significantly higher than the posttest score (*p*<.001) and follow-up score (*p*<.001); 2) the posttest score was significantly lower than the pretest score (*p*<.001) and follow-up score (*p*<.05); and 3) the follow-up score was significantly lower than the pretest score (*p*<.001), but significantly higher than the posttest score (*p*<.05).

^7^Bonferroni Pairwise Comparisons indicated that: 1) the pretest score was significantly higher than the posttest score (*p*<.001), but not significantly different than the follow-up score (*p*=1.00); 2) the posttest score was significantly lower than the pretest score (*p*<.001) and the follow-up score (*p*<.001); and 3) the follow-up score was not significantly different than the pretest score (*p*=1.00), but was significantly higher than the posttest score (*p*<.001).

^8^Bonferroni Pairwise Comparisons indicated that: 1) the pretest score was significantly higher than the posttest score (*p*<.001), but not significantly different than the follow-up score (*p*=.22); 2) the posttest score was significantly lower than the pretest score (*p*<.001) and the follow-up score (*p*<.001); and 3) the follow-up score was not significantly different than the pretest score (*p*=.22), but was significantly higher than the posttest score (*p*<.001).

**Supplementary Table 3**

**Repeated Measures MANOVA Examining Behavioral Outcome Variables (*n*=155)**

¹Bonferroni Pairwise Comparisons indicated that: 1) the pretest score was significantly lower than the posttest score (*p*<.001) and follow-up score (*p*<.001); 2) the posttest score was significantly higher than the pretest score (*p*<.001), but did not differ significantly from the follow-up score (*p*=.17); and 3) the follow-up score was significantly higher than the pretest score (*p*<.001), but did not differ significantly from the posttest score (*p*=.17).

²Bonferroni Pairwise Comparisons indicated that: 1) the pretest score was significantly lower than the posttest score (*p*<.001) and follow-up score (*p*<.001); 2) the posttest score was significantly higher than the pretest score (*p*<.001) and follow-up score (*p*<.01); and 3) the follow-up score was significantly higher than the pretest score (*p*<.001), but significantly lower than the posttest score (*p*<.01).

^3^Bonferroni Pairwise Comparisons indicated that: 1) the pretest score was significantly lower than the posttest score (*p*<.01), but did not differ significantly from the follow-up score (*p*=1.00); 2) the posttest score was significantly higher than the pretest score (*p*<.01) and follow-up score (*p*<.001); and 3) the follow-up score was not significantly different from the pretest score (*p*=1.00), but was significantly lower than the posttest score (*p*<.001).

^4^Bonferroni Pairwise Comparisons indicated that: 1) the pretest score was significantly higher than the posttest score (*p*<.001) and follow-up score (*p*<.001); 2) the posttest score was significantly lower than the pretest score (*p*<.001), but did not differ significantly from the follow-up score (*p*=1.00); and 3) the follow-up score was significantly lower than the pretest score (*p*<.001), but was not significantly different than the posttest score (*p*=1.00).

^5^Bonferroni Pairwise Comparisons indicated that: 1) the pretest score was significantly higher than the posttest score (*p*<.05) and follow-up score (*p*<.05); 2) the posttest score was significantly lower than the pretest score (*p*<.05), but did not differ significantly from the follow-up score (*p*=.96); and 3) the follow-up score was significantly lower than the pretest score (*p*<.05), but did not differ significantly from the posttest score (*p*=.96).
